# IRE1α-XBP1 Affects the Mitochondrial Function of Aβ25–35-Treated SH-SY5Y Cells by Regulating Mitochondria-Associated Endoplasmic Reticulum Membranes

**DOI:** 10.3389/fncel.2021.614556

**Published:** 2021-03-25

**Authors:** Bingcong Chu, Maoyu Li, Xi Cao, Rulong Li, Suqin Jin, Hui Yang, Linlin Xu, Ping Wang, Jianzhong Bi

**Affiliations:** Department of Neurology, Second Hospital of Shandong University, Jinan, China

**Keywords:** Alzheheimer's disease, amyloid-beta-protein, IRE1α-XBP1, cytotoxicity, mitochondria impairment, mitochondria associated ER membranes

## Abstract

**Background:** Neurotoxicity induced by the amyloid beta (Aβ) peptide is one of the most important pathological mechanisms of Alzheimer's disease (AD). Activation of the adaptive IRE1α-XBP1 pathway contributes to the pathogenesis of AD, making it a potential target for AD therapeutics. However, the mechanism of IRE1α-XBP1 pathway involvement in AD is unclear. We, therefore, investigated the effect of the IRE1α-XBP1 axis in an *in vitro* AD model and explored its potential mechanism.

**Methods:** The human neuroblastoma cell line, SH-SY5Y, was used. Cells were treated with Aβ25–35, with or without 4μ8c, an inhibitor of IRE1α. Cells were collected and analyzed by Western blotting, quantitative real-time PCR, electron microscopy, fluorescence microscopy, calcium imaging, and other biochemical assays.

**Results:** Aβ-exposed SH-SY5Y cells showed an increased expression of XBP1s and p-IRE1α. 3-(4,5-dimethylthiazol-2-yl)-2,5-diphenyltetrazolium bromide (MTT) and calcium imaging analysis showed that the IRE1α inhibitor, 4μ8c, reduced Aβ-induced cytotoxicity. Increased levels of ATP, restoration of mitochondrial membrane potential, and decreased production of mitochondrial reactive oxygen species after Aβ treatment in the presence of 4μ8c showed that inhibiting the IRE1α-XBP1 axis effectively mitigated Aβ-induced mitochondrial dysfunction in SH-SY5Y cells. Furthermore, Aβ treatment increased the expression and interaction of IP3R, Grp75, and vdac1 and led to an increased endoplasmic reticulum (ER)–mitochondria association, malfunction of mitochondria-associated ER-membranes (MAMs), and mitochondrial dysfunction. These deficits were rescued by inhibiting the IRE1α-XBP1 axis.

**Conclusion:** These findings demonstrate that Aβ peptide induces the activation of the IRE1α-XBP1 axis, which may aggravate cytotoxicity and mitochondrial impairment in SH-SY5Y cells by targeting MAMs. Inhibition of the IRE1α-XBP1 axis provides the protection against Aβ-induced injury in SH-SY5Y cells and may, therefore, be a new treatment strategy.

## Introduction

Alzheimer's disease (AD), the most common cause of dementia, is a chronic and progressive neurodegenerative disease. A typical pathological hallmark of AD is senile plaques, which are composed primarily of amyloid beta (Aβ) peptides. Aβ peptides are produced by the proteolytic cleavage of the amyloid precursor protein (APP) by β-secretase and γ-secretase cleavage (Thinakaran and Koo, 2008). Several studies have reported that APP and γ-secretase exist in mitochondrial–endoplasmic reticulum (ER) lipid raft structures [mitochondria-associated ER membranes (MAMs)], indicating that Aβ is mainly produced at MAMs (Schreiner et al., 2015; Del Prete et al., 2017).

The ER takes part in protein synthesis, post-translational modifications, and protein folding. Under physiological conditions, ER stress (ERS) occurs when the accumulation of unfolded or misfolded proteins exceeds the processing capacity of the ER, which activates the unfolded protein response to reassert cell homeostasis (Deegan et al., 2013; Cai et al., 2016). Inositol-requiring enzyme 1α (IRE1α), a transmembrane protein that has both serine/threonine-protein kinase and endoribonuclease activities, is a major ER sensor that responds to unfolded protein response signals (Chen and Brandizzi, 2013). IRE1α is autophosphorylated upon activation into phosphorylated IRE1α (p-IRE1α) and excises a 26-base intron from the X-box binding protein 1 (XBP1) mRNA to produce an active transcription factor (XBP1s) (Tam et al., 2014). Several studies have demonstrated the activation of the IRE1α-XBP1 pathway in AD, e.g., p-IRE1α and XBP1s are elevated in different regions of the human brain of patients affected with AD (Hoozemans et al., 2009; Lee et al., 2010). Xu et al. have shown that, after Aβ treatment, IRE1α induces XBP1 splicing and increases the XBP1 levels (Xu et al., 2019). Moreover, there is evidence that the activation of IRE1α aggravates the histopathological progress of AD (Duran-Aniotz et al., 2017). These studies suggest that IRE1α-XBP1 signaling may participate in causing AD; however, its specific role in the disease remains poorly defined.

Mitochondria perform multiple functions, such as energy production, metabolic regulation, signal transduction regulation, and calcium buffering (Lin and Beal, 2006). Abnormal mitochondrial function affects the production and toxicity of Aβ. Mitochondrial dysfunction can promote APP cleavage to produce Aβ (Pavlov et al., 2011), which accumulates in mitochondria, activates mitochondrial fission (Manczak et al., 2011), and increases the mitochondrial calcium levels to further impair mitochondrial function (Calvo-Rodriguez et al., 2020). A vicious circle that contributes to the onset and progression of AD is created by mitochondrial dysfunction. Song et al. (2018) found that IRE1 α-XBP1 inhibits mitochondrial respiration in ovarian cancer T cells. However, the effect of IRE1α on mitochondrial function in AD is not clear.

Based on the distinct roles of the ER and mitochondria, the impacts of their dysfunction in AD have largely been considered and studied independently. However, these organelles physically and functionally interact with and can regulate the function of each other (Rowland and Voeltz, 2012). MAMs, lipid raft regions where the endoplasmic reticulum has physical contact with mitochondria, play an important role in lipid synthesis, calcium homeostasis, and apoptosis signal transduction (Perrone et al., 2020). MAM disorders have recently gained attention in the pathogenesis and pathological process of AD. Changes in the behavior of MAMs may be related to various disordered functions in AD, such as Aβ production changes; mitochondrial damage; endoplasmic reticulum stress; and lipid, glucose, and calcium metabolism changes (Schreiner et al., 2015; Area-Gomez et al., 2018; van Vliet and Agostinis, 2018). Recent studies have shown that IRE1α interacts with inositol 1,4,5-trisphosphate receptor (IP3R) on MAMs to regulate calcium transfer from the ER to mitochondria (Malli and Graier, 2019).

In this study, we used Aβ25-35-treated SH-SY5Y cells to build a cell model of AD, which has been extensively used by researchers (Masci et al., 2015; Shekhar et al., 2018; Chen et al., 2019). The analysis of Aβ25–35-treated SH-SY5Y cells revealed the activation of the IRE1α-XBP1 axis. Based on these results, multiple approaches were employed to explore the effect of inhibiting the IRE1α-XBP1 axis on cell viability and mitochondrial function in the context of AD and to estimate the underlying mechanism. Overall, the results uncovered that the inhibition of IRE1α-XBP1 axis by an *in vitro* model of AD protects mitochondrial function by regulating MAM, which will inform the prevention and treatment of AD.

## Materials and Methods

### Cell Culture

Human neuroblastoma SH-SY5Y cells were obtained from the Cell Center of the Chinese Academy of Medical Sciences (Beijing, China). SH-SY5Y cells were propagated in Dulbecco's Modified Eagle's Medium (DMEM) (Biological Industries, Kibbutz Beit-Haemek, Israel) supplemented with 10% fetal bovine serum (Gibco, Carlsbad, CA, USA), 1% penicillin, and 1% streptomycin (Gibco, Carlsbad, CA, USA) at 37°C in humidified 5% CO_2_ and 95% air for 24 h prior to treatment. Aβ25–35 (MedChemExpress, Shanghai, China) was dissolved in double-distilled water, adjusted to 1 mM/L, and aggregated at 37°C for 5 days. The IRE1α inhibitor, 7-hydroxy-4-methyl-2-oxo-2H-1-benzopyran-8-carboxaldehyde (4μ8c) (MedChemExpress), was dissolved in dimethyl sulfoxide (DMSO, Solarbio, Beijing, China) to make a stock solution of 20 mM/L, and aliquots were stored at −20°C until use.

### Cell Viability Assay

Cell viability was determined by 3-(4,5-dimethylthiazol-2-yl)-2,5-diphenyltetrazolium bromide (MTT) assays. Cells were plated at 1 × 10^4^ cells per well in 96-well-plates and cultured at 37°C in a culture medium until a single layer of cells covered the bottom of the wells. Cells were then pre-treated with 4μ8c for 6 h and exposed to Aβ25–35 for another 24 h. After treatment, 5 mg/ml MTT (Solarbio) was added to each well, and the plates were incubated for 4 h. Subsequently, 200 μl DMSO was added to dissolve the formazan crystals. The absorbance was measured at 570 nm with a microplate reader (Multiskan MK3, Thermo Fisher Scientific, MA, USA). Experiments were repeated at least 3 times.

### Western Blotting

To extract proteins, cells were incubated in RIPA lysis buffer (Boster, Wuhan, China) supplemented with a complete protease inhibitor cocktail (Beyotime, Shanghai, China) for 30 min on ice. Supernatants were collected after centrifugation (12,000 × *g*, 4°C, 15 min), and protein concentrations were measured using a Pierce BCA Protein Assay Kit (Thermo Fisher Scientific). Protein samples were mixed with a loading buffer and heated to 95°C for 5 min to denature proteins. Protein samples were separated by electrophoresis on 8 ~ 10% SDS-PAGE gels. For SDS-PAGE, 20 micrograms of total protein was loaded per lane. Under low-temperature conditions, the proteins were transferred to polyvinylidene difluoride (PVDF) membranes (Millipore, Billerica, MA, USA) at a constant current of 200 mA using the wet transfer method, adjusting the required transfer time according to protein molecular weight: 10× Transfer buffer: Add 116 g of Tris Base and 24 g of Glycine to 1 L of double-distilled water and 1× Transfer buffer: 700 ml double distilled water, 200 ml methanol, and 100 ml 10× Transfer buffer. Membranes were blocked with 5% skimmed milk and incubated with primary antibody against XBP1 (Abcam, Cambridge, UK), p-IRE1α (Bioss, Beijing, China), IRE1α (Cell Signaling Technology, Inc. MA, USA), IP3R (Santa Cruz, CA, USA), Grp75 (Cell Signaling Technology), VDAC1 (Cell Signaling Technology), or β-actin (Proteintech, Wuhan, China) overnight at 4°C, followed by incubation with a horseradish peroxidase-conjugated secondary antibody (Boster) for 1 h at room temperature. After visualization using an ECL chemiluminescence kit (Merck Millipore) and a chemiluminescence imaging system (Protein Simple, USA), bands were quantified using Image J software (v1.8.0 http://imagej.nih.gov/ij/).

### Quantitative Real-Time PCR (qRT-PCR)

Total RNA was extracted from cells using TRIzol reagent (TaKaRa, Dalian, Liaoning, China). Total RNA concentration was determined using an ultraviolet spectrophotometer. Reverse transcription was conducted using a Prime Script™ RT Master Mix Kit (TaKaRa, Dalian, Liaoning, China) according to the instructions of the manufacturer. RT-PCR was performed using a SYBR Green qPCR Mix (Sparkjade, Qingdao, China) and an ABI ViiA™ 7 System. Specific primers for β-actin and XBP1 (Table 1) were generated by BioSune (Shanghai, China). The results were analyzed using the 2^−Δ*ΔCT*^ method. Data were expressed as the levels of mRNA of interest normalized to β*-actin* mRNA levels in each sample.

**Table 1:** Oligonucleotide primer sets for quantitative real-time PCR (RT-PCR).

**Name**	**Sequence (5^**′**^-3^**′**^)**	**Length**	**Tm**
XBP1 F	CTGAGTCCGCAGCAGGTG	18	59.46
XBP1 R	GGCTGGTAAGGAACTGGGTC	20	59.50
β-Actin F	CATGTACGTTGCTATCCAGGC	21	57.6
β-Actin R	CTCCTTAATGTCACGCACGAT	21	55.6

### Ca^2+^ Imaging

SH-SY5Y cells were loaded with Fura-2/AM (2 μM; Molecular Probes, Eugene, OR, USA) for 30 min at 37°C in an atmosphere of 95% O_2_ and 5% CO_2_. Fura-2/AM was dissolved in the Hank's balanced salt solution (HBSS) containing (in mM) 138 NaCl, 5 KCl, 0.3 KH_2_PO_4_, 4 NaHCO_3_, 2 CaCl_2_, 1 MgCl_2_, 10 HEPES, and 5.6 glucose, pH 7.4. Ca^2+^ imaging was performed as described previously (Zhang et al., 2011). The cell slide was incubated in the fluorescent calcium probe Fura-2/AM solution at 37°C for 30 min, placed on a calcium imaging epifluorescence microscope (Nikon Eclipse Ti), and continuously perfused with HBSS. Bradykinin (BK) is a common inflammatory factor whose receptor sensitivity and expression levels are closely related to cell apoptosis. We used 1 μM BK (Sigma) to examine the BK sensitivity of all SH-SY5Y groups. Thapsigargin 200 μM was used to estimate the calcium content of the ER. Fura-2/AM was excited with ultraviolet light alternately at 340 and 380 nm, and the fluorescence emission was detected at 510 nm using a computer-controlled monochromator. Wavelength selection, the timing of excitation, and the acquisition of images were controlled using the Metafluor Imaging System (Molecular Devices, Sunnyvale, CA). Digital images were stored for off-line analysis. The ratio of the fluorescence signal measured at 340 nm divided by the fluorescence signal measured at 380 nm was used to measure the increase in intracellular Ca^2+^.

### Measurement of MMP

Mitochondrial membrane potential was determined by assaying tetramethylrhodamine (TMRM, Thermo Fisher Scientific) according to the protocol of the manufacturer. After drug stimulation, SH-SY5Y cells were incubated with 200 nM TMRM at 37°C for 30 min and then washed 2 times with PBS. Images were captured using a LSM 700 laser scanning confocal microscope (Zeiss). The photos were taken with 10× eyepiece and 20× objective lens, the scale bar was 50 μm, and the total length of the picture was 280 μm. Regarding the quantitative assessment of fluorescence signal intensity, the threshold was applied and Image J software was used to measure automatically according to the method described by Ververis et al. (2016). Also, scale bars were added using ImageJ software. Images shown were globally adjusted for brightness and contrast, but individual portions of images were not modified in any way.

### Measurement of Mitochondrial Reactive Oxygen Species

Mitochondrial ROS activity was measured using MitoSOX Red (Invitrogen, Carlsbad, CA, USA), a redox-sensitive fluorescent probe that selectively targets mitochondria. Cells were incubated with 5 μM MitoSOX Red for 30 min at 37°C and washed 3 times with DMEM. Subsequently, the cells were fixed with 4% paraformaldehyde for 30 min, after which they were washed 3 times with PBS. Cells were then stained with DAPI, and images of the slides were captured using a fluorescence microscope (OLYMPUS BX43). The photos were taken with 10× eyepiece and 20× objective lens, the scale bar was 50 μm, and the total length of the picture was 280 μm. Three wells were used for each group. Experiments were repeated 3 times.

### Measurement of ATP

Adenosine triphosphate content was determined using an ATP assay kit (Beyotime). Briefly, cells were lysed by a cellular ATP-releasing reagent on ice and centrifuged at 12,000 × *g* for 15 min at 4°C. Supernatants were added to a mixture of luciferin-luciferase according to the instructions of the manufacturer. ATP was measured using a GloMax™ 20/20 luminometer (Promega).

### Transmission Electron Microscopy

Transmission electron microscopy was used to observe MAM structures within SH-SY5Y cells. ER–mitochondria contact sites were defined as sites with a distance of <30 nm between the membranes of the two organelles (Rowland and Voeltz, 2012). Harvested cells were placed in 2.5% glutaraldehyde at 4°C overnight, stained with osmic acid, and then dehydrated in alcohol and acetone. Subsequently, cells were embedded in epoxy resin, sectioned at 70 nm using an ultramicrotome (Leica, Wetzlar, Germany), and stained with 2% uranium acetate and lead citrate. TEM images were captured using a Hitachi HT7800 at 80.0 kV (Hitachi Co., Tokyo, Japan). The ER–mitochondria contact length in the given distance (30 nm) ranges between the membranes was measured using ImageJ software, and statistical analyses were performed using GraphPad Prism7.

### Calcium Concentration Analysis

The cytoplasmic Ca^2+^-sensitive fluorescent dye, Fura-2/AM (Molecular Probes), and the mitochondrial Ca^2+^-sensitive fluorescent dye, Rhod-2/AM (AAT Bioquest, Sunnyvale, CA, USA), were used to determine Ca^2+^ concentration according to the instructions of the manufacturer. After drug treatment, cells were incubated with Fura-2/AM or Rhod-2/AM for 30 min at 37°C. The cell samples were then analyzed by fluorescence microscopy (OLYMPUS BX43). The photos were taken with 10× eyepiece and 20× objective lens, the scale bar was 50 μm, and the total length of the picture was 280 μm. All experiments were performed in triplicate.

### Co-immunoprecipitation Assays

After drug treatment, cells were lysed on ice in an immunoprecipitation buffer (Beyotime) with a protease inhibitor cocktail (Roche, Basel, Switzerland). One-fifth of the cell lysates were prepared as input samples, and the rest was used for coimmunoprecipitation. Cell lysates were pre-cleared with Protein A Sepharose beads for 1 h, and the supernatant was incubated with a primary antibody of IP3R (Santa Cruz) at 4°C overnight. The Protein A Sepharose beads were then added to the system and incubated for 6 h at 4°C. After incubation, the beads were washed 3 times with cold PBS. The immunoprecipitates were subjected to Western blotting analysis with an anti-Grp75 antibody or anti-VDAC1 antibody (Cell Signaling Technology).

### Cell Transfection of Small Interfering RNA

IRE1α siRNA was produced by GenePharma (GenePharma, Shanghai, China) and transfected using transfection reagent siRNA-mate (GenePharma, Shanghai, China) according to the protocol of the manufacturer. The sequence were shown as follows: 5′-CUCCGAGCCAUGAGAAAUATT-30 (sense) and 5′-UAUUUCUCAUGGCUCGGAGTT-30 (antisense).

### Statistical Analysis

All data were expressed as mean ± SD. A one-way ANOVA or the two-tailed Student's *t*-test was used to determine statistical significance. Data were analyzed using Prism 7.00 (GraphPad, San Diego, USA) or SigmaPlot 13.0 (Systat Software Inc.). The *p* < 0.05 was considered statistically significant.

## Results

### Activation of the IRE1α Signaling Pathway in Aβ-Treated SH-SY5Y Cells

**Figure 1:**
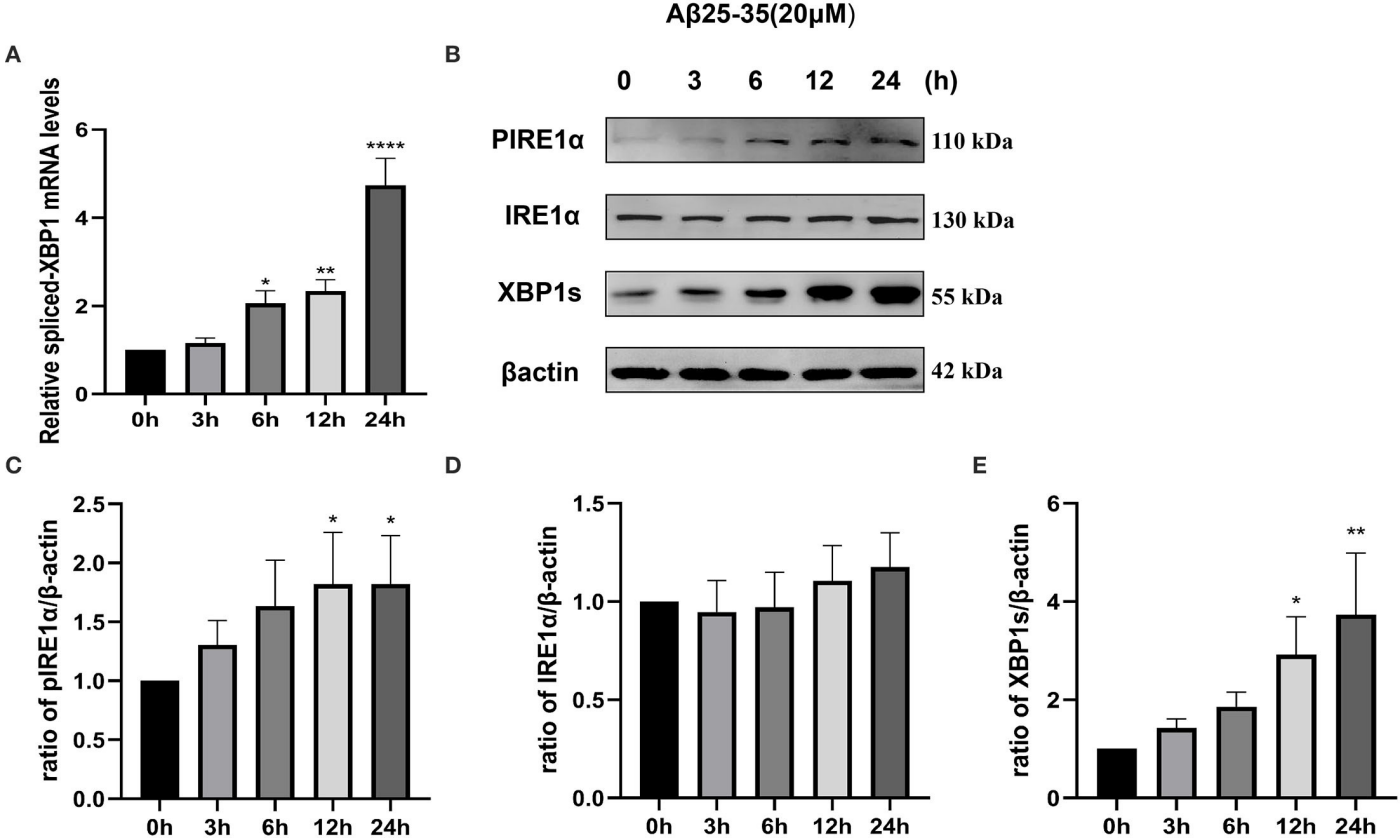
Activation of the IRE1 signaling pathway in Aβ-treated SH-SY5Y cells. Cells were treated with 20 μM Aβ25–35 for 0, 3, 6, 12, and 24 h. **(A)** Quantitative real-time PCR (qRT-PCR) analysis of spliced-*XBP1* mRNA levels. Data were expressed as the fold-change compared with β*-actin* mRNA levels. **(B)** Western blot analysis of pIRE1α, IRE1α, and XBP1s proteins. **(C–E)** Densitometric analysis of pIRE1α, IRE1α, and XBP1s protein levels normalized to the β-actin level. The data are presented as mean ± SD (*n* = 3), *P < 0.05 compared with the control. **P < 0.01 compared with the control. ****P < 0.0001 compared with the control.

To investigate whether the IRE1α signaling pathway is activated in SH-SY5Y cells after Aβ25–35 exposure, we determined the activation of IRE1α by measuring IRE1α phosphorylation and expression of spliced XBP1, a downstream factor of IRE1α. As shown in Figure 1A, SH-SY5Y cells were treated with 20 μM Aβ25–35 for 3, 6, 12, and 24 h. Spliced-*XBP1* mRNA levels were upregulated (*P*_6h_ = 0.0269; *P*_12h_ = 0.0054; *P*_24h_ < 0.0001). Although the protein expression level of IRE1α did not change significantly (P>0.05), its phosphorylated form, pIRE1α (*P*_12h_ = 0.0409; *P*_24h_ = 0.0407), and the downstream molecule, XBP1s (*P*_12h_ = 0.0195; *P*_24h_ = 0.002; Figures 1B–D), was increased in a time-dependent manner, indicating that the IRE1α pathway was activated after Aβ25–35 treatment.

### The Effect of IRE1α-XBP1 Activation on the Viability of Aβ-Treated Cells

**Figure 2:**
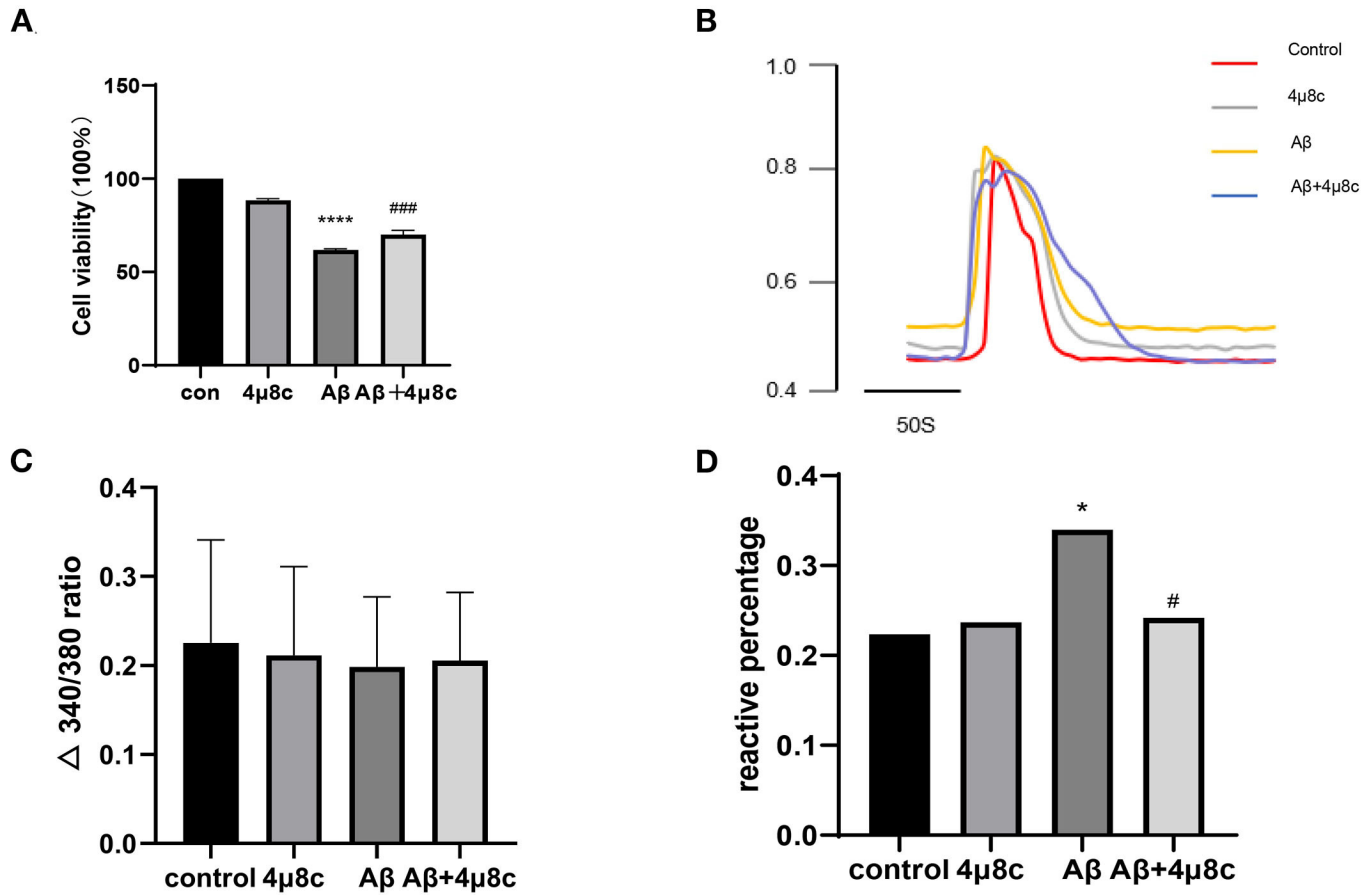
The effect of the IRE1α-XBP1 activation on the viability of Aβ-treated cells. Cells were pretreated with or without 20 μM 4μ8c for 6 h, followed by treatment with 20 μM Aβ25–35 for 24 h. **(A)** Cell viability was measured using the 3-(4,5-dimethylthiazol-2-yl)-2,5-diphenyltetrazolium bromide (MTT) assay. **(B)** Representative trace of Ca^2+^ upon 1 μM bradykinin (BK) stimulation. **(C)** The amplitude of 1 μM BK-evoked Ca^2+^ increase was calculated as the difference between the peak and the baseline. **(D)** The number of cells that responded to BK was quantitatively analyzed and is expressed as a percentage of the response. The data are presented aes mean ± SD, *P < 0.05 compared with the control. ****P < 0.0001 compared with the control. ^#^*P* < 0.05 compared with the Aβ25–35-alone group. ^###^*P* < 0.001 compared with the Aβ25–35-alone group.

To determine whether IRE1α-XBP1 is involved in Aβ-induced death of SH-SY5Y cells, we blocked IRE1α with various inhibitors, such as 4μ8C, STF-083010, and MKC-3946 (Zhang et al., 2014; Li et al., 2019). The data show that these inhibitors of IRE1α can protect SH-SY5Y cells against Aβ-induced injury in a concentration-dependent manner (Supplementary Figure 1). Because of the similar pharmacological effects of these inhibitors, we chose 4μ8c as a representative. Then, each group was pretreated with 4μ8C (20 μM) for 6 h and with 20 μM Aβ for 24 h. As shown in Figure 2A, Aβ25–35 decreased the cell viability while pretreatment with 4μ8C partially reversed this effect (*P* < 0.001).

Later, we evaluated the functional status of cells using calcium imaging. BK plays an important role in apoptosis by regulating the intracellular Ca^2+^ levels (Hong et al., 2004; Ran et al., 2005); therefore, we reasoned that Aβ treatment might cause a similar phenomenon in SH-SY5Y cells. A typical reaction of SH-SY5Y cells to BK is shown in Figure 2B. Aβ-treated SH-SY5Y cells (34.0%, *n* = 206) had a higher sensitivity to BK than control cells (22.4%, *n* = 170), while 4μ8c (24.2%, *n* = 244) could provide relief to the injury (*P* = 0.028, Figure 2D), although there was no effect on the amplitude of intracellular calcium increase (*P* > 0.05, Figure 2C). 4μ8C pretreatment partly prevented the increase in sensitivity to BK without changing the amplitude of calcium increase.

In addition, we used the siRNA to knockdown the IRE1α-XBP1 pathway and validated the protein expressive level (Supplementary Figures 2A–D) for further cell viability analysis in Aβ25–35-treated SH-SY5Y cells. MTT assay analysis indicated that the knockdown of the IRE1α-XBP1 pathway reduced Aβ25–35-induced cell injury (85.69 ± 12.06 vs. 67.86 ± 3.52; *P* = 0.017, Supplementary Figure 2E).

### The Effect of IRE1α-XBP1 on Mitochondrial Function of Aβ-Treated Cells

Mitochondria are crucial for cell survival, and Aβ exposure affects various aspects of mitochondrial function (Pagani and Eckert, 2011). Therefore, we further explored whether the IRE1α-XBP1 pathway affects Aβ-induced mitochondrial dysfunction through the measurement of ATP content, MMP depolarization levels, and mitochondrial ROS.

#### The Effect of IRE1α-XBP1 on ATP Content of Aβ-Treated Cells

**Figure 3:**
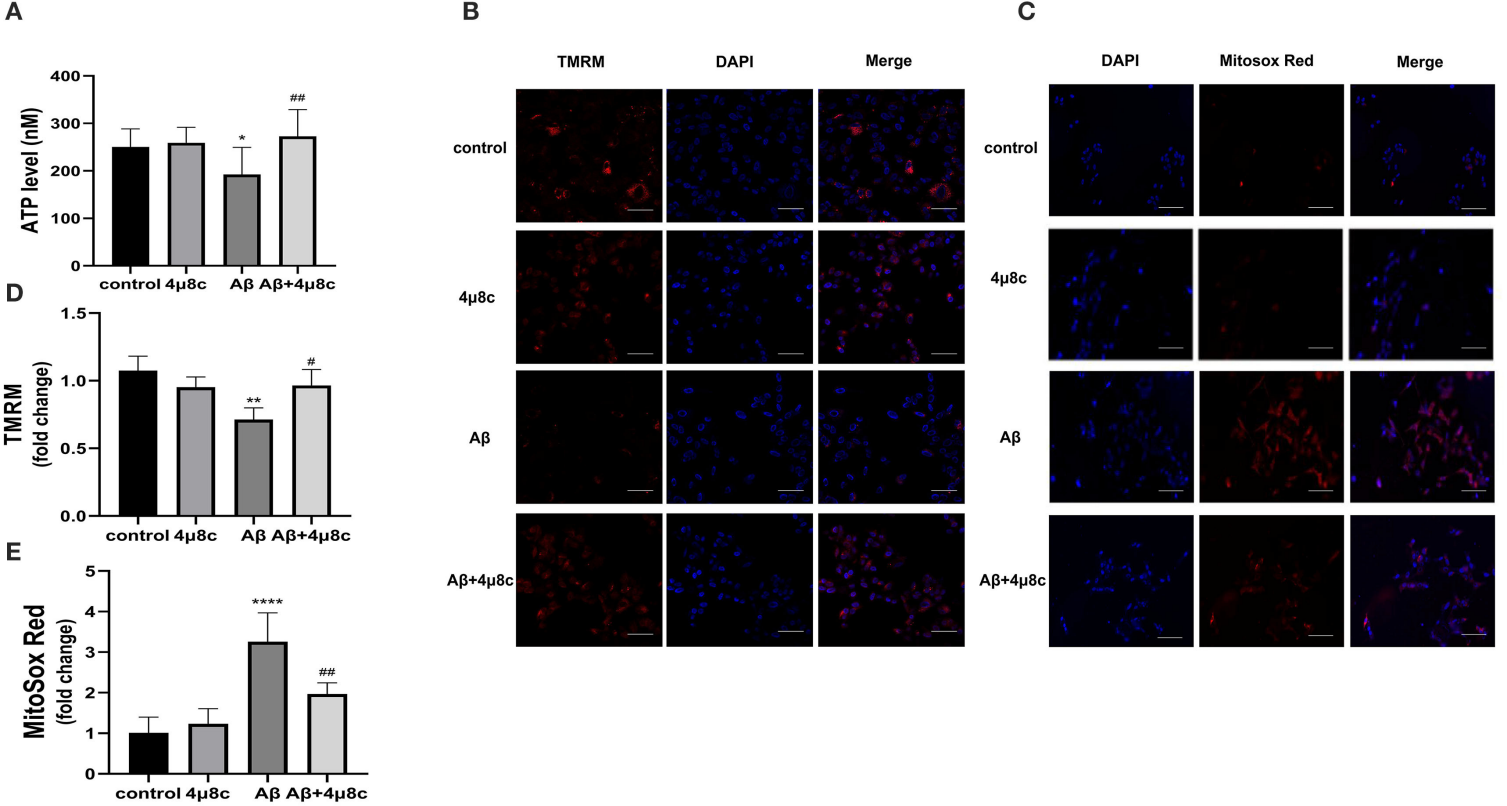
The effect of IRE1α-XBP1 on mitochondrial function in Aβ-treated cells. Cells were pretreated with or without 20 μM 4μ8c for 6 h, followed by treatment with 20 μM Aβ25–35 for 24 h. **(A)** Cells were lysed on ice with an ATP kit to measure the ATP levels. **(B)** Mitochondrial membrane potential (MMP) was examined using the fluorescent probe, tetramethylrhodamine (TMRM). **(C)** Mitochondrial reactive oxygen species (ROS) levels were determined using MitoSoX. Scale bar = 50 μm. **(D)** The mean fluorescence intensity of each group was stained by TMRM. **(E)** Mean fluorescence intensity of each group was stained by MitoSoX. The data are presented as mean ± SD (*n* = 3), *P < 0.05 compared with the control. **P < 0.01 compared with the control. ****P < 0.0001 compared with the control. ^#^*P* < 0.05 compared with the Aβ25–35-alone group. ^##^*P* < 0.01 compared with the Aβ25–35-alone group.

The levels of ATP significantly declined after the treatment of SH-SY5Y cells with Aβ (192.4 ± 57.26 vs. 250.5 ± 38.19 nM; *P* = 0.028), but pretreatment with 4μ8C effectively increased the ATP levels in Aβ-treated SH-SY5Y cells (272.9 ± 56.17 vs. 192.4 ± 57.26 nM; *P* = 0.002; Figure 3A).

#### The Effect of IRE1α-XBP1 on the Mitochondrial Potential of Aβ-Treated Cells

The mitochondrial membrane potential is critical for ATP production; therefore, we assessed the change in mitochondrial potential (ΔΨm) by staining with TMRM. Compared with the control group, cells treated with Aβ alone showed lower levels of the TMRM signal (0.71 ± 0.09 vs. 1.08 ± 0.11; *p* = 0.0027). However, 4μ8C substantially rescued the loss of TMRM signal in Aβ-treated cells (0.97 ± 0.12 vs. 0.71 ± 0.09; *p* = 0.0296; Figure 3B).

Furthermore, we assessed the effect of the knockdown of the IRE1α-XBP1 pathway on MMP. Consistently, we observed that Aβ25–35 treatment induced the loss of MMP (0.70 ± 0.07 vs. 1.01 ± 0.14; *p* = 0.0021), which was alleviated after the suppression of the IRE1α-XBP1 pathway (0.93 ± 0.09 vs. 0.70 ± 0.07; *p* = 0.0115, Supplementary Figure 3).

#### Effect of IRE1α-XBP1 on ROS of Aβ-Treated Cells

Reactive oxygen species play a critical role in mitochondria dysfunction. We, therefore, assessed the levels of mitochondrial ROS using MitoSOX Red, which is a redox-sensitive mitochondrial dye. As presented in Figure 3C, Aβ substantially increased mitochondrial ROS production (3.26 ± 0.71 vs. 1.01 ± 0.39; *p* < 0.0001), but, in the presence of 4μ8C, Aβ-treated cells produced significantly less superoxide (1.97 ± 0.27 vs. 3.26 ± 0.71; *p* = 0.0025).

### The Effect of IRE1α-XBP1 on MAMs in Aβ-Treated Cells

Alterations in ER–mitochondria crosstalk are closely related to mitochondrial dysfunction (Filadi et al., 2017). Therefore, we investigated whether IRE1α-XBP1 impacted mitochondrial function by mediating alterations in MAMs.

#### The Effect of IRE1α-XBP1 on the Formation of ER–Mitochondria Contact Points

**Figure 4:**
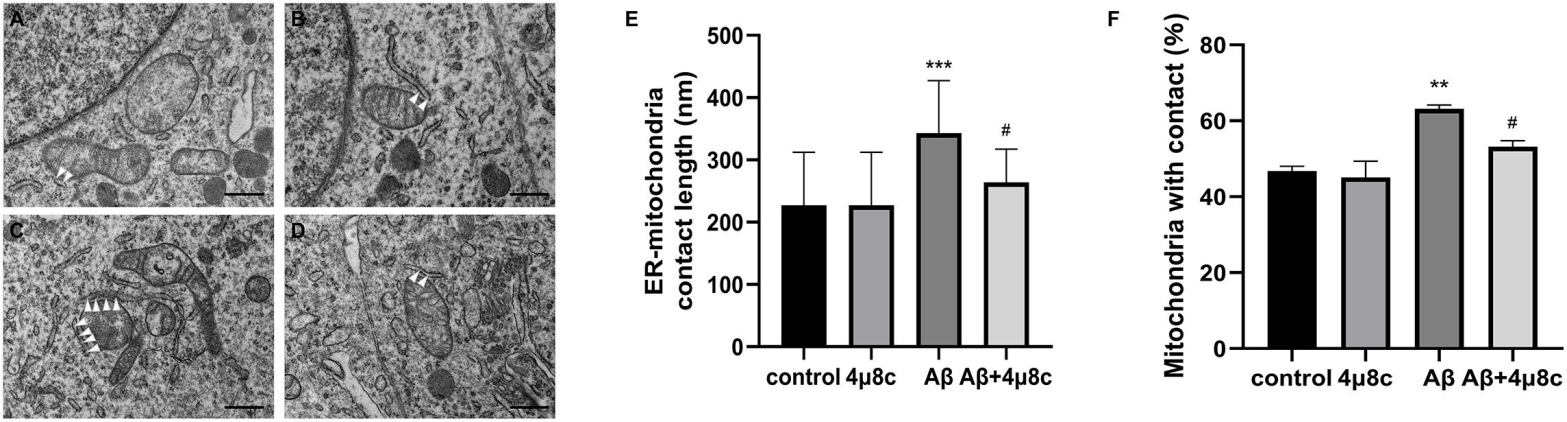
The effect of IRE1α-XBP1 on the length of endoplasmic reticulum (ER)–mitochondria contact sites in Aβ-treated cells. Cells were pretreated with or without 20 μM 4μ8c for 2 h, followed by treatment with 20 μM Aβ25–35 for 4 h. **(A–D)** Representative electron micrographs of ER–mitochondria contact sites in **(A)** the control group, **(B)** the 4μ8c group, **(C)** the Aβ group, and **(D)** the Aβ+4μ8c group cells. The white triangles indicate ER–mitochondrial contact sites. Scale bar =0.5 μm. **(E)** Quantitative analysis of average length of the ER–mitochondria association. **(F)** Bar graphs show the percentage of mitochondria in contact with ER. (control-72 mitochondria, 4μ8c-79 mitochondria, Aβ-83 mitochondria, Aβ+4μ8c-75 mitochondria). The data are presented as mean ± SD (*n* = 3), **P < 0.01 compared with the control. ***P < 0.001 compared with the control. ^#^*P* < 0.05 compared with the Aβ25–35-alone group.

First, we examined whether the IRE1α-XBP1 pathway alters the status of ER–mitochondria coupling in Aβ25–35 treated cells. Electron microscopy revealed that both the length of the ER–mitochondria contact sites (343.3 ± 84.13 vs. 227.3 ± 84.98 nm; *P* < 0.001) and the percentage of mitochondria in contact with ER (63.18 ± 0.96 vs. 46.75 ± 1.3; *P* = 0.008) were increased by Aβ25–35 treatment. However, this phenomenon was significantly reduced when cells were pretreated with 4μ8c (*P*_lenth_ = 0.017, *P*_percentage_ = 0.046), while 4μ8c treatment alone did not alter ER–mitochondrial interactions (*P* > 0.05; Figure 4).

#### The Effect of IRE1α-XBP1 on Calcium Transfer Between the ER and Mitochondria

**Figure 5:**
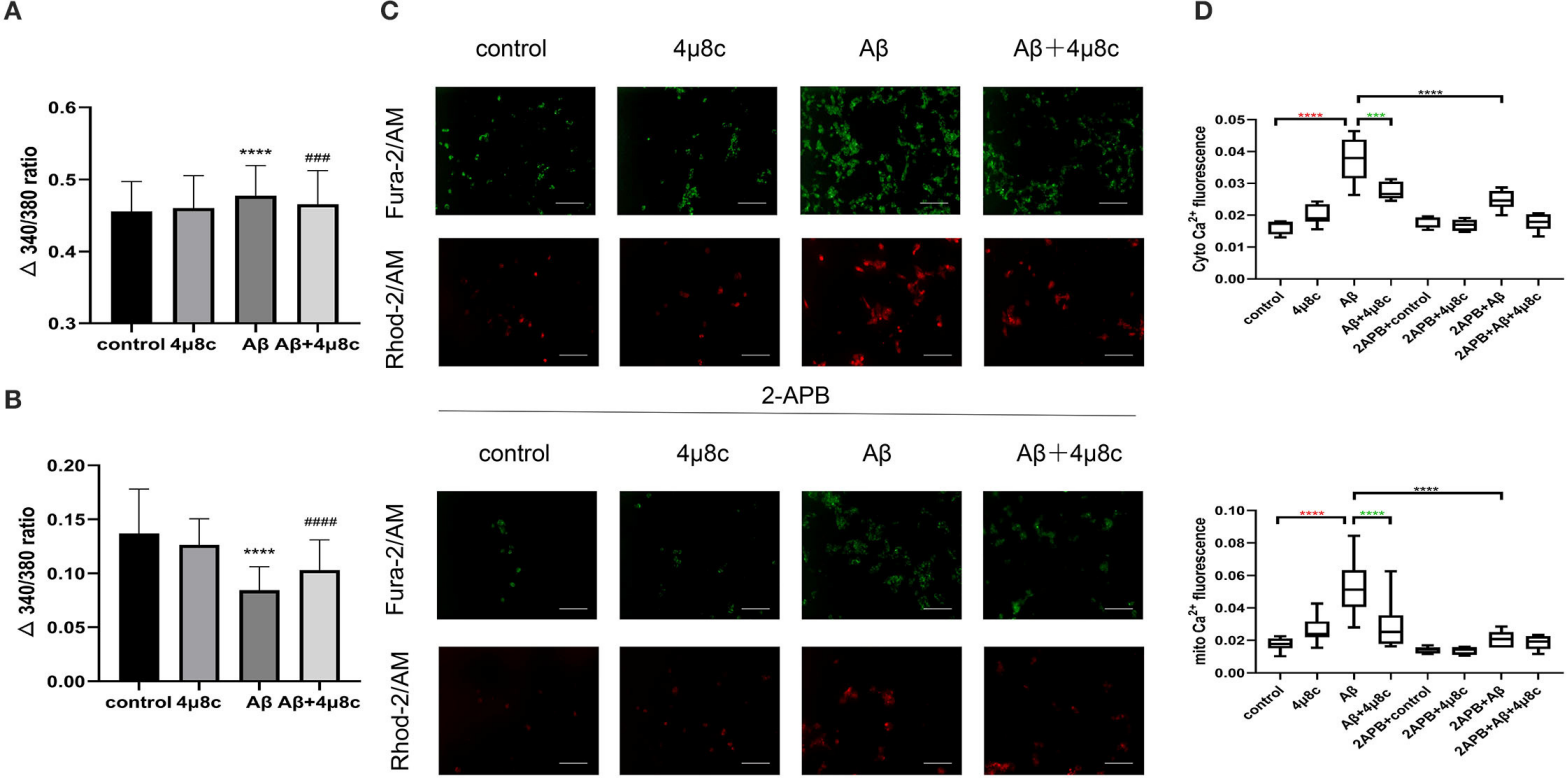
The effect of IRE1α-XBP1 on calcium transfer between the ER and mitochondria in Aβ-treated cells. Cells were pretreated with or without 20 μM 4μ8c for 6 h, followed by treatment with 20 μM Aβ25–35 for 24 h. **(A)** Basal calcium levels in cells were measured using Fura-2 ratios. **(B)** Quantification of Tg-induced Ca^2+^ release (reflecting ER Ca^2+^ content). ****p < 0.0001 compared with the control group. ^###^*P* < 0.001 compared with the Aβ25-35-alone group. ^####^*P* < 0.0001 compared with the Aβ25–35-alone group. **(C)** Cytoplasmic and mitochondrial Ca^2+^ content in cells measured by Fura-2/AM and Rhod-2/AM, respectively. Scale bar = 50 μm. **(D)** The quantification of cytoplasmic calcium and mitochondrial calcium fluorescence intensity. Red * indicates the comparison of the control group with the Aβ25–35 alone group. Green * indicates the comparison of the Aβ25–35 alone group with the Aβ+4μ8c group. Black * indicates the comparison of the Aβ25–35 alone group with the 2APB+Aβ25–35 group. The data are presented as mean ± SD (*n* = 3).

The MAM function was tested by evaluating ER–mitochondria calcium transfer. Then, basal intracellular calcium was examined using the calcium-sensitive dye, Fura-2, and it was higher in Aβ-treated cells compared with the control group (0.484 ± 0.0428 vs. 0.445 ± 0.037, *P* < 0.0001), while 4μ8c reduced Aβ-induced basal calcium levels (0.466 ± 0.047 vs. 0.484 ± 0.0428, *P* < 0.001; Figure 5A). Next, the calcium concentration of the ER was assessed indirectly using thapsigargin, which inhibits the sarco(endo)plasmic reticulum Ca^2+^-ATPase (SERCA) activity, resulting in the depletion of ER calcium due to a lack of calcium refilling. As shown in Figure 5B, the calcium content of the ER was significantly lower in Aβ-treated cells compared with that in control cells (0.084 ± 0.022 vs. 0.137 ± 0.041, *P* < 0.0001). However, 4μ8c pretreatment partially restored the calcium content of the ER in Aβ-treated cells (0.103 ± 0.028 vs. 0.084 ± 0.022, *p* < 0.001). The relative cytosolic and mitochondrial Ca^2+^ levels in cells using the Fura-2/AM and Rhod-2/AM indicators, respectively, was assessed. Aβ25–35 treatment induced higher cytosolic and mitochondrial Ca^2+^ levels in SH-SY5Y cells compared with the control group (*P*_cytoCa2+_ < 0.0001, *P*_mitoCa2+_ < 0.0001), which was partially prevented by 4μ8c (*P*_cytoCa2+_ = 0.0002, *P*_mitoCa2+_ < 0.0001; Figure 5C, upper panel). To demonstrate that cytosolic and mitochondrial Ca^2+^ accumulation derives from the ER, the IP3R chelator, 2-APB, was applied to cells. Fluorescence microscopy revealed that 2-APB inhibited cytosolic and mitochondrial Ca^2+^ accumulation induced by Aβ (*P*_cytoCa2+_ < 0.0001, *P*_mitoCa2+_ < 0.0001; Figure 5C, lower panel). These results revealed that IRE1α was involved in Aβ25-35-induced ER–mitochondrial interactions in SH-SY5Y cells.

#### The Effect of IRE1α-XBP1 on the IP3R-Grp75-VDAC1 Complex in Aβ-Treated Cells

**Figure 6:**
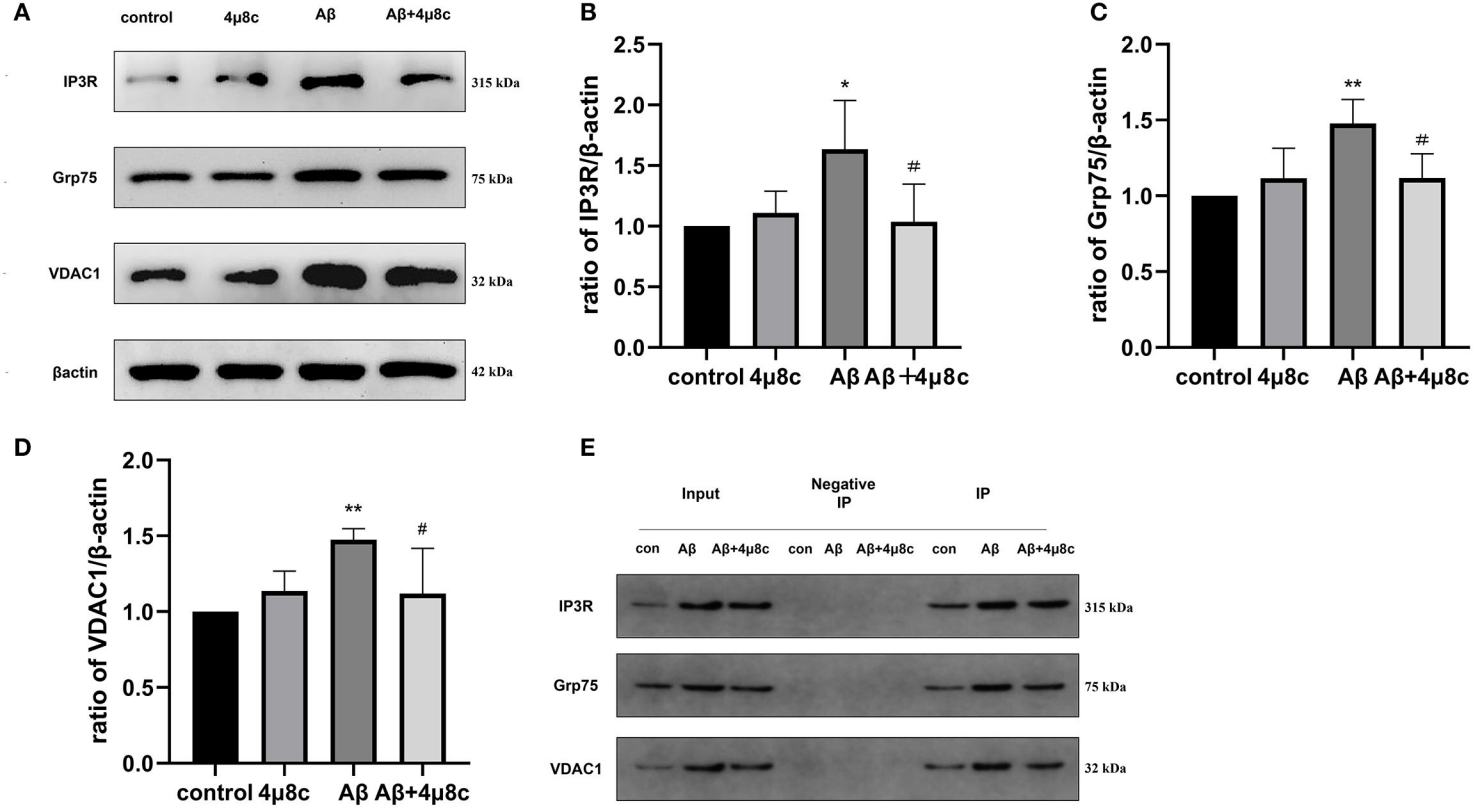
The effect of IRE1α-XBP1 on the IP3R-Grp75-VDAC1 complex in Aβ-treated cells. Cells were pretreated with or without 20 μM 4μ8c for 6 h, followed by treatment with 20 μM Aβ25–35 for 24 h. **(A)** Western blot analysis of IP3R, Grp75, and VDAC1 proteins. **(B–D)** Densitometric analysis of the protein levels of IP3R, Grp75, and VDAC1 normalized to the level of β-actin. **(E)** Co-immunoprecipitation (Co-IP) was performed to analyze the IP3R-Grp75-VDAC1 interaction. Normal rabbit IgG without antigenicity was used as a negative control. Lysates from cells in each group without immunoprecipitation were used as a positive control (input). The proteins pulled down by anti-IP3Rantibodies were analyzed by Western blotting. The data are presented as mean ± SD (*n* = 3), *p < 0.05 compared with the control. **p < 0.01 compared with the control. ^#^*P* < 0.05 compared with the Aβ25–35-alone group.

Previous studies have demonstrated that IRE1α interacts with IP3R and has a structural role at MAMs as a scaffold (Carreras-Sureda et al., 2019). IP3R is physically associated with the voltage-dependent anion channel, VDAC1, through Grp75 to form a tethered complex on MAMs, which regulates ER–mitochondria tethering and calcium signaling. We further investigated whether the effects of the IRE1α pathway on MAM behavior are mediated through IP3R-Grp75-VDAC1. Western blot analysis indicated that Aβ induced higher levels of IP3R (*P* = 0.0269), Grp75 (*P* = 0.0035), and VDAC1 (*P* = 0.0099) in SH-SY5Y cells compared with the control group, while IP3R (*P* = 0.038), Grp75 (*P* = 0.024), and VDAC1 (*P* = 0.047) levels were markedly decreased in SH-SY5Y cells pretreated with 4μ8c. In addition, Co-IP experiments revealed that the interaction among the IP3R-Grp75-VDAC1 complex was increased after Aβ treatment. However, in the presence of 4μ8c, the interaction of the IP3R-Grp75-VDAC1 complex in Aβ-treated cells was reduced (Figure 6). These results indicated that Aβ-exposure changes the participation of IRE1α in MAM behavior in association with the IP3R-Grp75-VDAC1 axis.

## Discussion

The roles of Aβ in the pathogenesis of AD have been extensively investigated and their importance in mitochondrial dysfunction in AD has becoming increasingly apparent. The IRE1α/XBP1 signaling pathway is a part of a cellular program that protects against ER stress (Ni et al., 2018), but it also plays a role in the development of AD. AD progression at the histopathological level is associated with chronic activation of IRE1α in the brain, and IRE1α deficiency significantly reduces the accumulation of Aβ in the brain of 5xFAD mice (Duran-Aniotz et al., 2017). Although IRE1α-XBP1 is activated in AD brains and is involved in AD pathogenesis, its effects on mitochondrial dysfunction in AD have not been investigated. Therefore, we need to clarify the effects of the IRE1α-XBP1 axis on mitochondrial dysfunction in Aβ-exposed cells and to determine the mechanism behind this effect. In the present study, the inhibition of IRE1α-XBP1 alleviated mitochondrial dysfunction in Aβ25–35-treated SH-SY5Y cells, which may be achieved by affecting MAMs.

Given that activation of IRE1α-XBP1 is closely related to AD pathology, we first investigated the effects of Aβ25–35 on the activation of IRE1α signaling by determining the levels of the phosphorylated form of IRE1α and spliced-XBP1. Aβ significantly increased the levels of p-IRE1α and spliced-XBP1 (Figure 1). These data were consistent with findings from others, suggesting that the IRE1α signaling pathway was activated in Aβ-treated cells (Pinkaew et al., 2015; Thummayot et al., 2016). The accumulation of Aβ peptide in the brain exerts neurotoxic effects and neuronal death (Hardy and Higgins, 1992). We tested the cell viability of SH-SY5Y cells treated by Aβ25–35 and 4μ8c to assess whether IRE1α signaling is involved in this process. The results show that inhibition of the IRE1α-XBP1 axis partially relieved Aβ-mediated cytotoxicity. The calcium imaging results further support this conclusion. Apoptotic processes can induce higher BK receptor sensitivity (Nokkari et al., 2015). When cells were stimulated with BK, Aβ was more sensitive to BK compared with cells of the control group, while the inhibition of the IRE1α-XBP1 axis blocked the effects of Aβ. These data indicate that Aβ-exposed cells are mostly in the apoptotic state and that inhibiting the IRE1α-XBP1 axis ameliorates this process (Figure 2). However, there was no statistically significant difference in the amplitude of the BK response among groups, although the Aβ group showed a lower mean amplitude than the control group. We believe that when the BK receptor was activated, not all calcium was released from the ER into the cytoplasm, which reduced the difference between groups caused by this phenomenon.

Increased mitochondrial dysfunction is evident in the AD brain and there is an association between Aβ and mitochondrial functions in AD. Aβ interferes with oxidative phosphorylation and ROS production within mitochondria, and decreases ΔΨm, complex IV (cytochrome c oxidase) activity, and the generation of ATP(Hauptmann et al., 2009). Our results of ATP levels, mitochondrial membrane potential, and mitochondrial ROS, showed mitochondrial impairment in Aβ25–35-treated cells. We also found that inhibiting the IRE1α-XBP1 axis could alleviate mitochondrial damage in Aβ25–35-treated cells (Figure 3). Moreover, the genetic inhibition of IRE1α-XBP1 axis was performed by RNA interference technique and further demonstrate that inhibiting IRE1α signaling might have therapeutic potential for AD by mediating the recovery of mitochondrial functions.

The sites of physical communication between mitochondria and the ER are defined as MAMs. It has been shown that MAM function and ER–mitochondrial communication are increased significantly in PS-knockout cells, in PS-knockdown cells, and in fibroblasts from both FAD and SAD patients (Area-Gomez et al., 2012). Notably, this increased MAM function correlated with a significantly increased area of apposition between ER and mitochondria (Schon and Area-Gomez, 2013). Consistently, an AD cell model was established by treatment with Aβ25–35 in our study, showing an increase in MAM contact and function. In addition, several studies have shown that MAMs are crucial for the efficient transmission of Ca^2+^ signals between the ER and mitochondria (Galmes et al., 2016). The transfer of Ca^2+^ from the ER to mitochondria is necessary for mitochondrial ATP production and cell survival. However, an excessive uptake of Ca^2+^ into mitochondria may trigger mitochondrial dysfunction, leading to the loss of mitochondrial membrane potential, resulting in decreased ATP synthesis and enhancement of mitochondrial ROS production (Penna et al., 2018). Interestingly, mitochondrial Ca^2+^ uptake mainly takes place through MAMs. Arruda et al. (2014) found that an increased MAM formation resulted in mitochondrial calcium overload, which, in turn, affected the mitochondrial function in obesity. In the present study, in Aβ-treated SH-SY5Y cells, the inhibition of the IRE1α-XBP1 axis caused a decrease in the physical and functional interactions between the ER and mitochondria at the contact sites, as demonstrated by two independent approaches, electron microscopy and measurement of calcium transferred between the ER and mitochondria (Figures 4, 5). The results indicate that inhibiting the IRE1α-XBP1 axis may have the potential to alleviate mitochondria dysfunction in Aβ-treated cells by modulating MAM integrity and calcium flux.

IP3R is the Ca^2+^ release channel located in the ER membrane that connects with VDAC1 through Grp75 to enable Ca^2+^ transfer from the ER to mitochondria. The IP3R-Grp75-VDAC1 complex is also proposed to act as a molecular tether, physically linking the ER to the mitochondria (Szabadkai et al., 2006). Therefore, we explored whether the alterations in calcium signaling observed in each treatment group were caused by changes in the level of the IP3R-Grp75-VDAC1 complex. Notably, Carreras-Sureda et al. (2019) showed that IRE1α localizes to the ER–mitochondria contact sites and interacts with IP3R to determine the MAM structure and MAM Ca^2+^ signaling. Data presented in this study showed that Aβ25–35 induced higher levels of expression of IP3R, Grp75, and VDAC1 and increased their interaction, while an IRE1α inhibitor reduced the effect of Aβ25–35 on this protein (Figure 6). All of the above data indicate that inhibiting IRE1α-XBP1 can mitigate mitochondria dysfunction in Aβ-treated cells, which is achieved by regulating the integrity and function of MAMs *via* the interaction with the IP3R3-Grp75-VDAC1 complex.

## Conclusions

**Figure 7:**
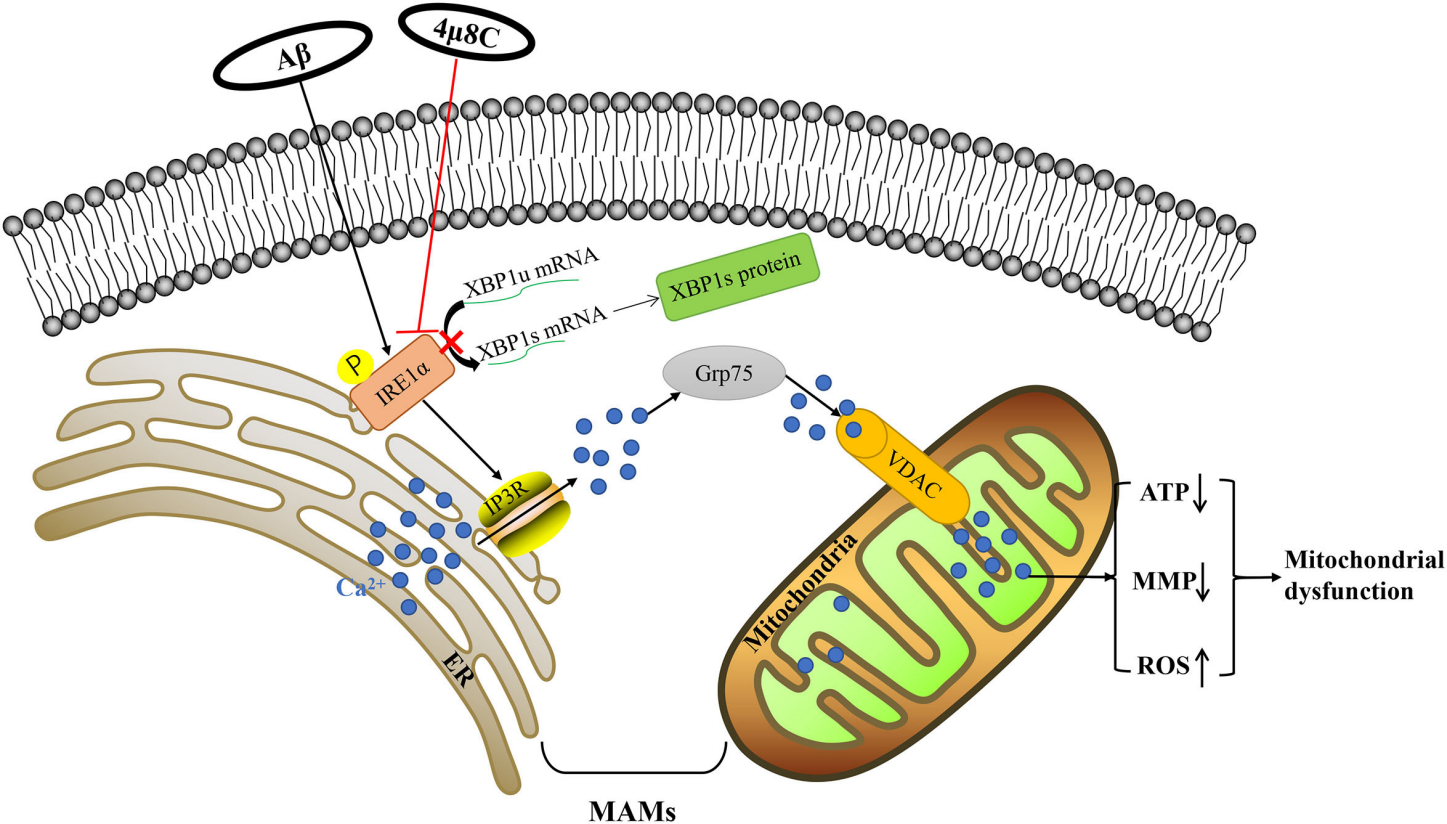
Schematic diagram for Aβ-induced mitochondrial dysfunction *via* the IRE1α-XBP1 pathway. Based on our experimental data, Aβ-induced activation of the IRE1α-XBP1 pathway facilitated the expression and interaction of IP3R, Grp75, and VDAC1, which further led to the increased the level of ER–mitochondria association and malfunction of mitochondria-associated ER-membranes (MAMs). Then, the increase of calcium ions transferred from the ER to the mitochondria *via* MAM leads to mitochondrial dysfunction. Inhibition of the IRE1α-XBP1 pathway ameliorated these Aβ25–35-induced changes *via* the regulation of MAM, which fostered neuroprotection.

We have demonstrated that altered calcium signaling through MAMs plays a fundamental role in connecting the IRE1α-XBP1 axis to mitochondrial dysfunction in Aβ-treated cells. Aβ enhanced the IP3R3-Grp75-VDAC1 complex, leading to increased ER–mitochondria association and calcium transfer and subsequent mitochondria dysfunction, but inhibiting the IRE1-XBP1 axis reversed this process (Figure 7). This study reveals that the inhibition of the IRE1-XBP1 axis is a potential strategy for developing AD therapeutics.

## Data Availability Statement

The original contributions presented in the study are included in the article/Supplementary Material, further inquiries can be directed to the corresponding author/s.

## Author Contributions

PW, JB, and BC contributed to the conception and study design. BC performed the experiments. ML, XC, RL, LX, SJ, and HY contributed to data collection and data analysis. PW, JB, and BC wrote the manuscript. All authors finally approved the manuscript before submission.

## Conflict of Interest

The authors declare that the research was conducted in the absence of any commercial or financial relationships that could be construed as a potential conflict of interest.
