# Supplementary material for: IRE1α-XBP1 Affects the Mitochondrial Function of Aβ25–35-Treated SH-SY5Y Cells by Regulating Mitochondria-Associated Endoplasmic Reticulum Membranes
Source: Front Cell Neurosci. 2021 Mar 25;15:614556. doi: 10.3389/fncel.2021.614556 (PMC8027129; doi:10.3389/fncel.2021.614556)
Supplement: Supplementary file 1 [file Data_Sheet_1.PDF]

Method：

Cell transfection of small interfering RNA (siRNA)

IRE1α siRNA was produced by GenePharma (GenePharma, Shanghai, China) and transfected using transfection reagent siRNA-mate (GenePharma) according to the manufacturers protocol. Sequences were as follows: 5’-CUCCGAGCCAUGAGAAAUATT-30(sense), 5’-UAUUUCUCAUGGCUCGGAGTT-30(antisense);

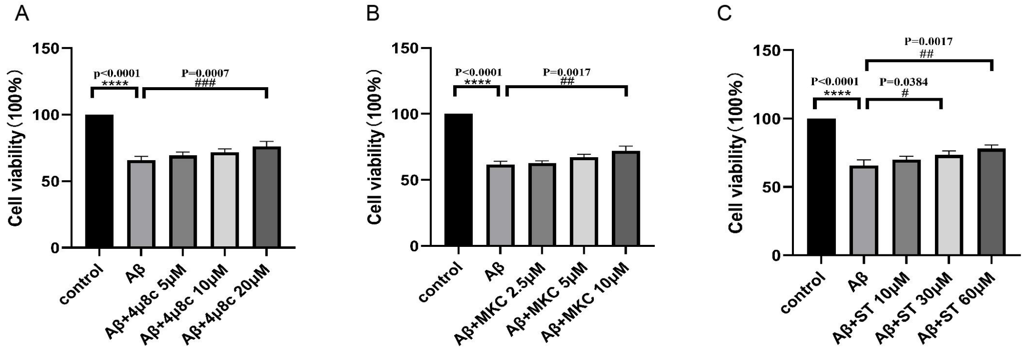


Fig S1: Effects of various IRE1α inhibitors on cell viability. The SH-SY5Y cells were pretreated with (**A**) 4μ8c (0, 5, 10, or 20 μM), (**B**) MKC-3946 (0, 2.5, 5, or 10 μM), (**C**) STF-083010 (0, 10, 30, or 60 μM) for 6 h followed by exposure to 20 μM Aβ25-35 for 24 h. Cell viability was measured using the MTT assay. The data are the mean ± SD (n=3), ****p<0.0001 compared with the control. #P<0.05 compared with the Aβ25–35-alone group. ##P<0.01 compared with the Aβ25–35-alone group. ###P<0.001 compared with the Aβ25–35-alone group.

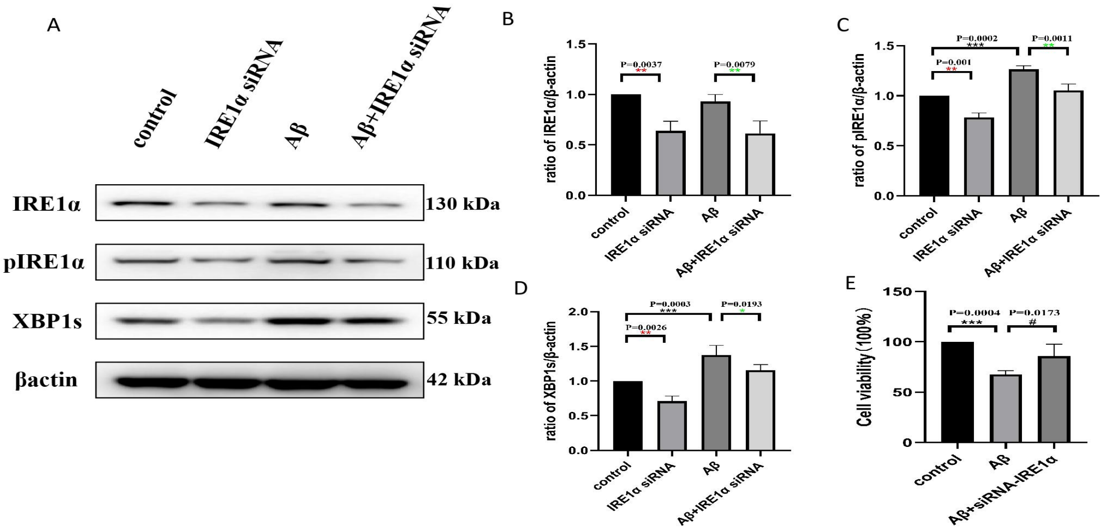

Fig S2: The effect of silencing IRE1α on cell viability in Aβ25-35-treated cells. (**A**) Representative immunoblots of IRE1α, pIRE1α, and XBP1s showed knockdown of IRE1α by the specific IRE1α siRNA. (**B**, **C** and **D**) Densitometric analysis of pIRE1α, IRE1α and XBP1s protein levels normalized to the β-actin level. Red indicates the comparison of the control group with the IRE1α siRNA group. Green * indicates the comparison of the Aβ25-35 alone group with the Aβ+IRE1α siRNA group. Black * indicates the comparison of the Aβ25-35 alone group with the control group. (**E**) Cell viability was measured using the MTT assay. The data are the mean ± SD (n=3).

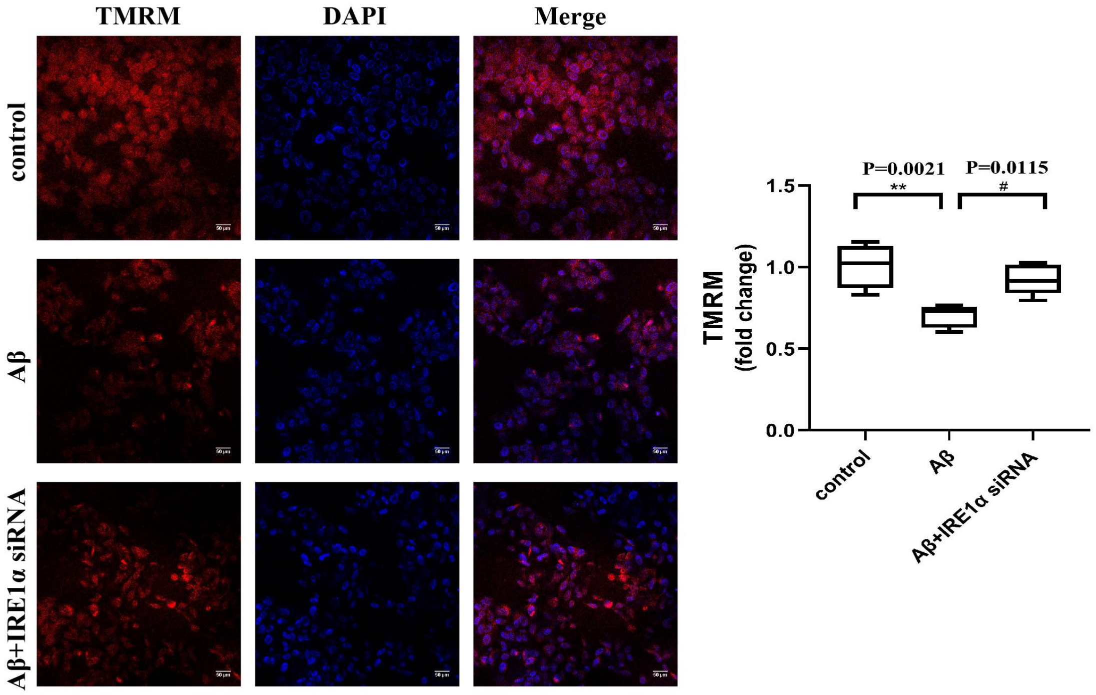


Fig S3: The effect of silencing IRE1α on mitochondrial membrane potential in Aβ25-35-treated cells. Mitochondrial membrane potential was examined using the fluorescent probe, TMRM.
