# Supplementary figures and images for: IRE1α-XBP1 Affects the Mitochondrial Function of Aβ25–35-Treated SH-SY5Y Cells by Regulating Mitochondria-Associated Endoplasmic Reticulum Membranes
Source: Front Cell Neurosci. 2021 Mar 25;15:614556. doi: 10.3389/fncel.2021.614556 (PMC8027129; doi:10.3389/fncel.2021.614556)

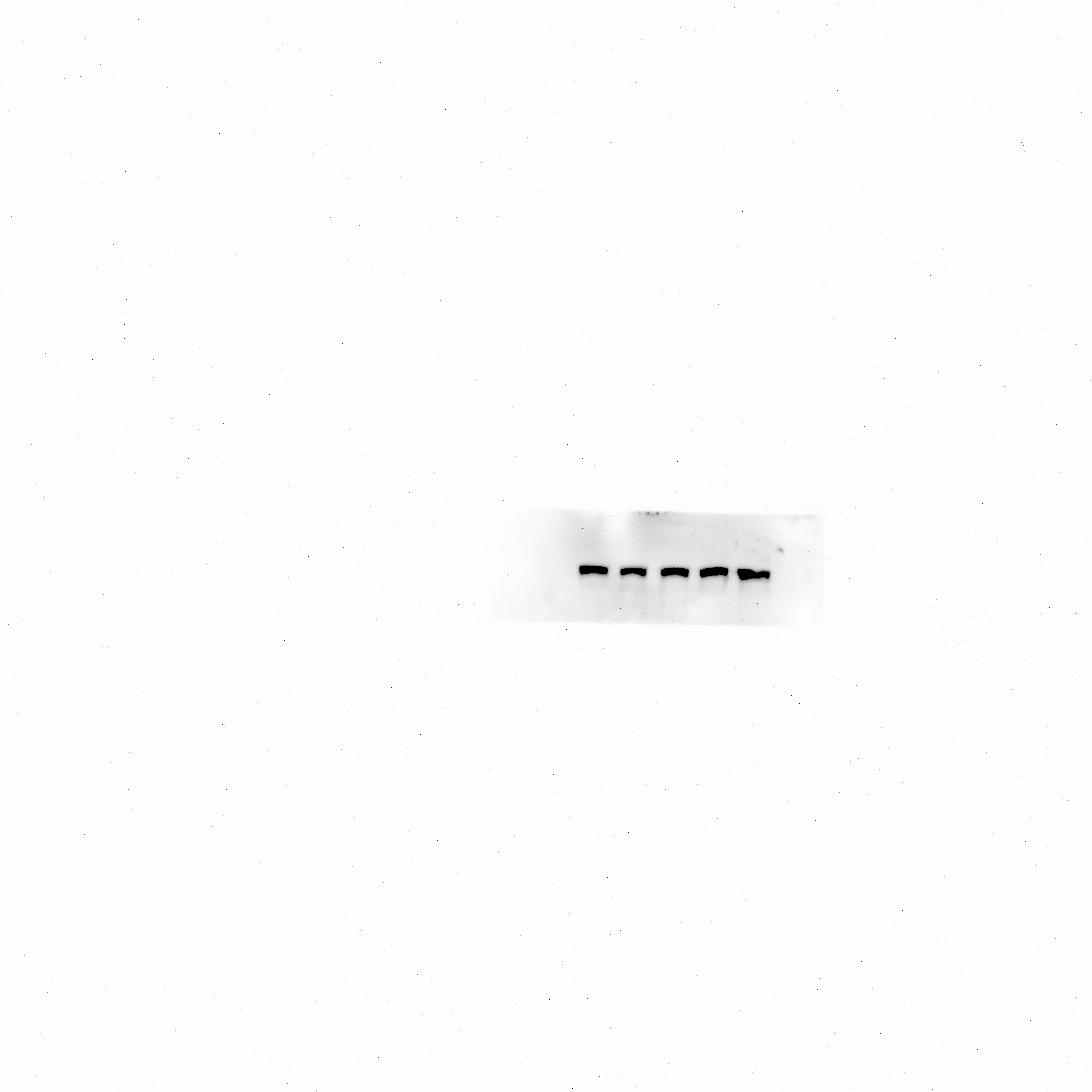

Supplement: Supplementary file 2 [file Image_1.TIF]

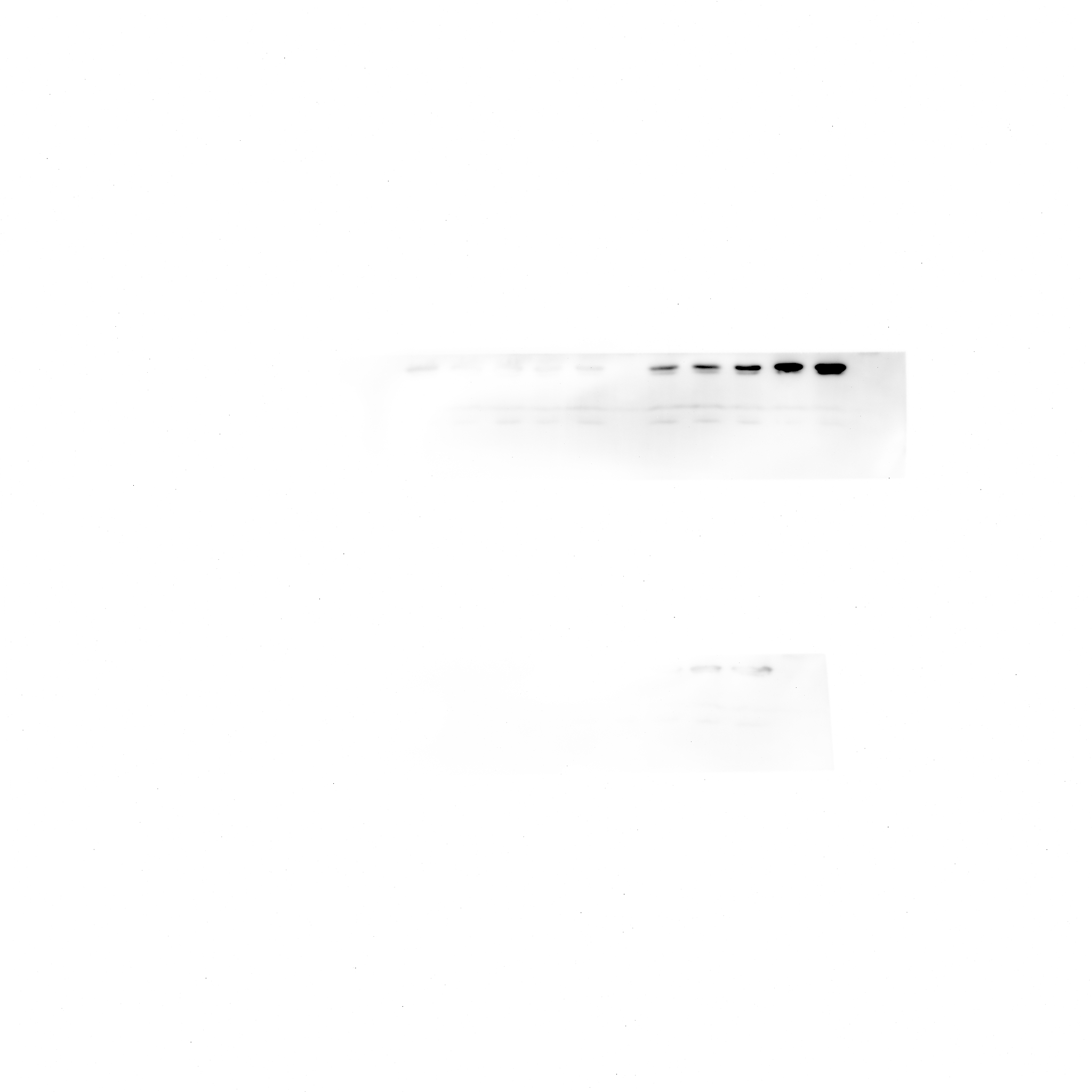

Supplement: Supplementary file 3 [file Image_2.TIF]

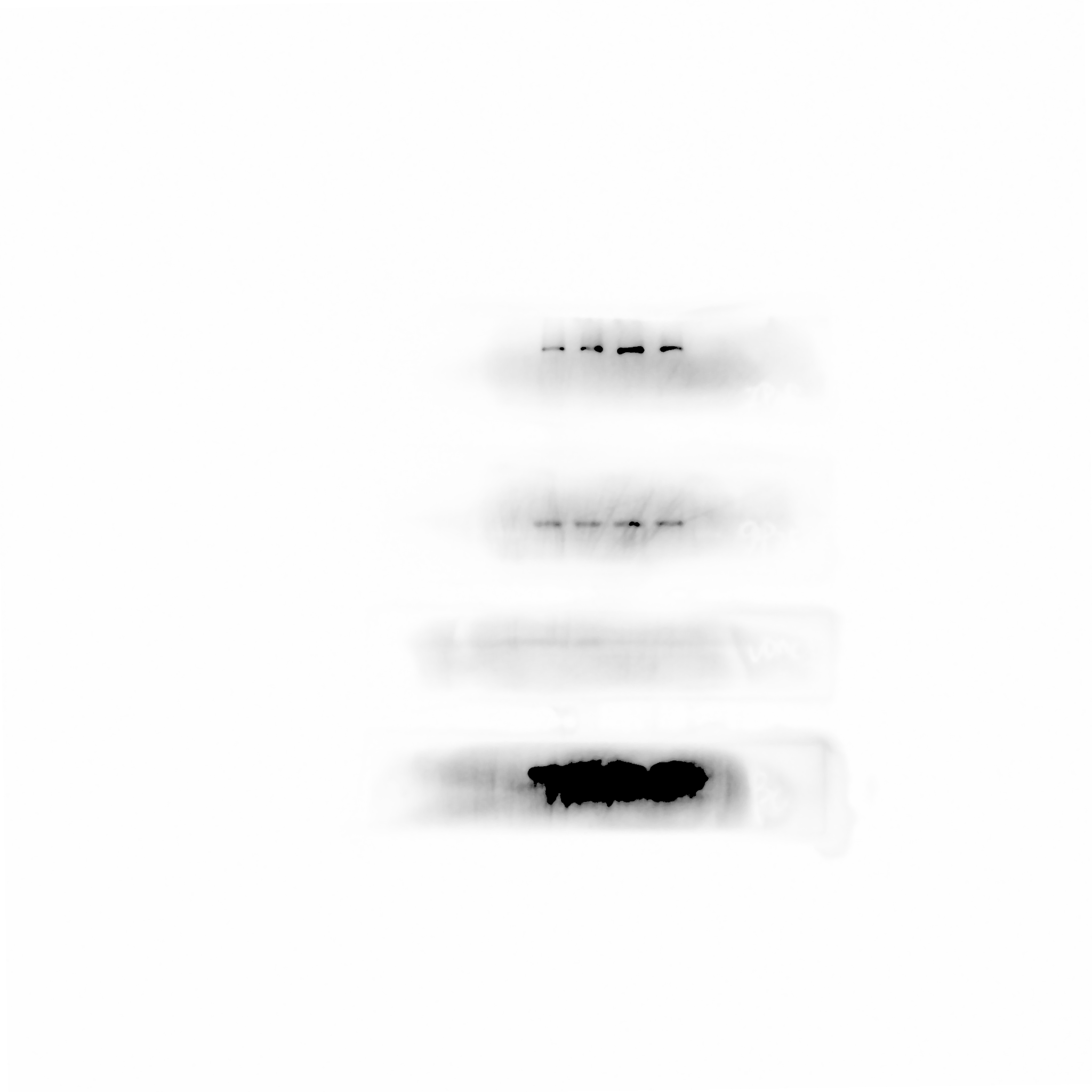

Supplement: Supplementary file 4 [file Image_3.TIF]

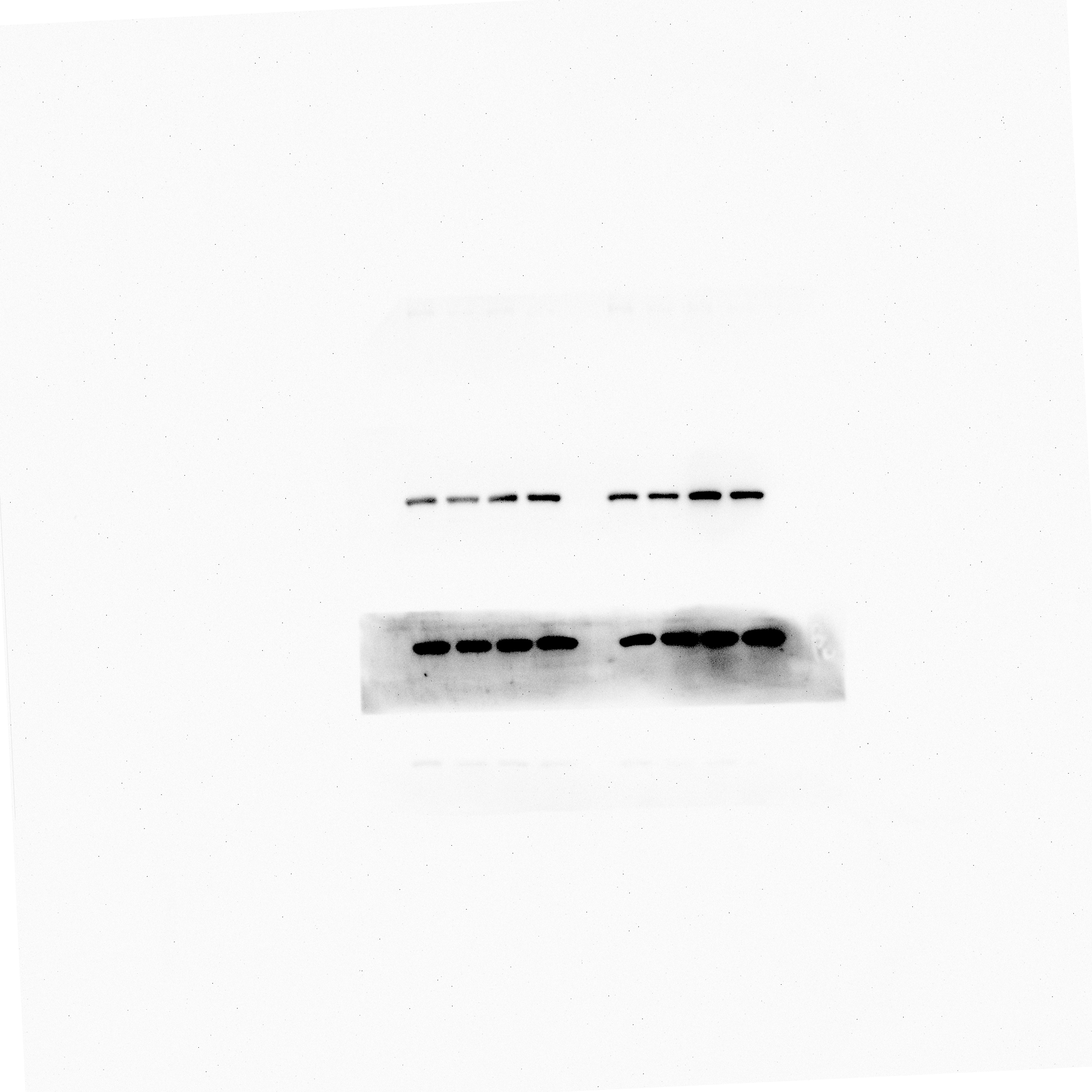

Supplement: Supplementary file 5 [file Image_4.TIF]

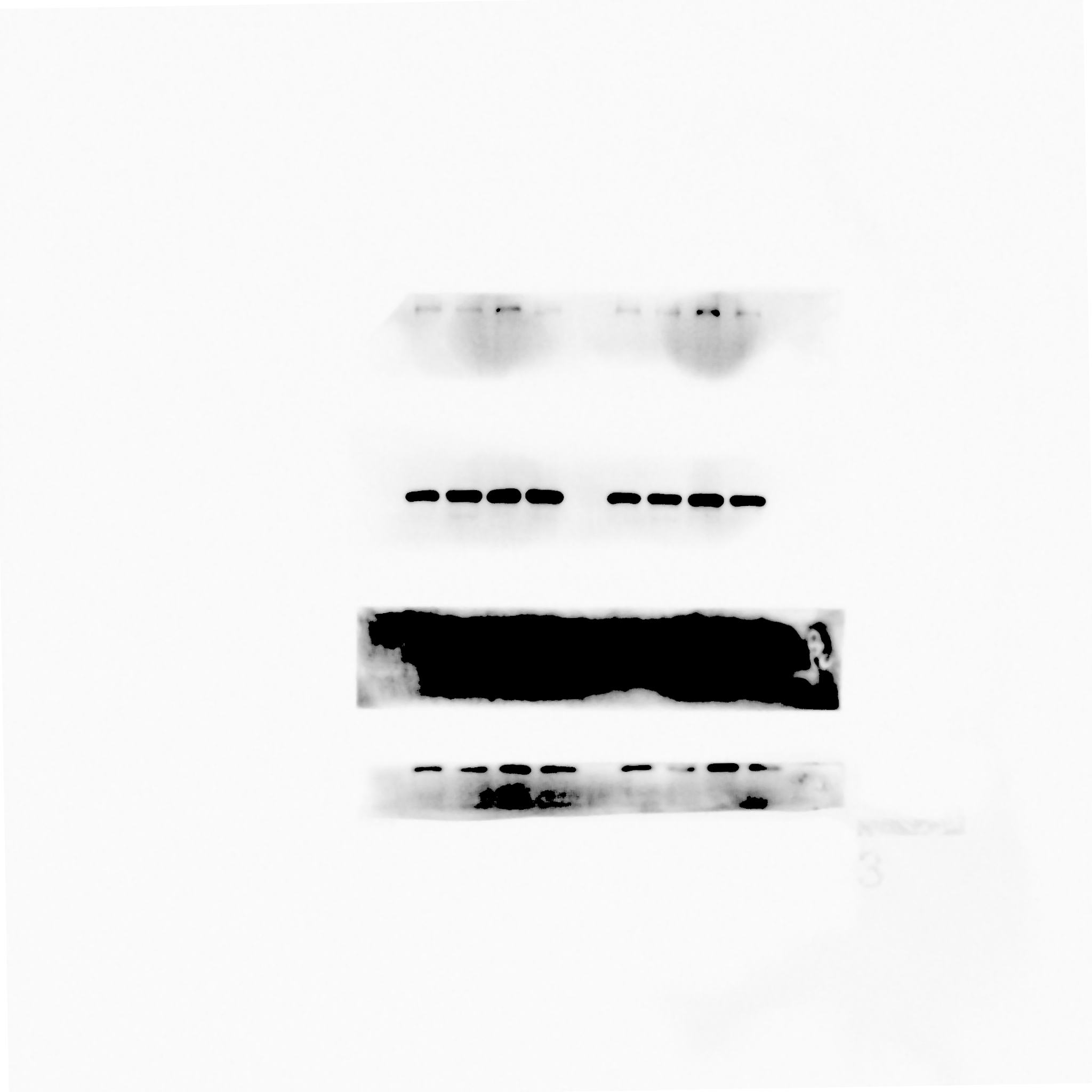

Supplement: Supplementary file 6 [file Image_5.TIF]

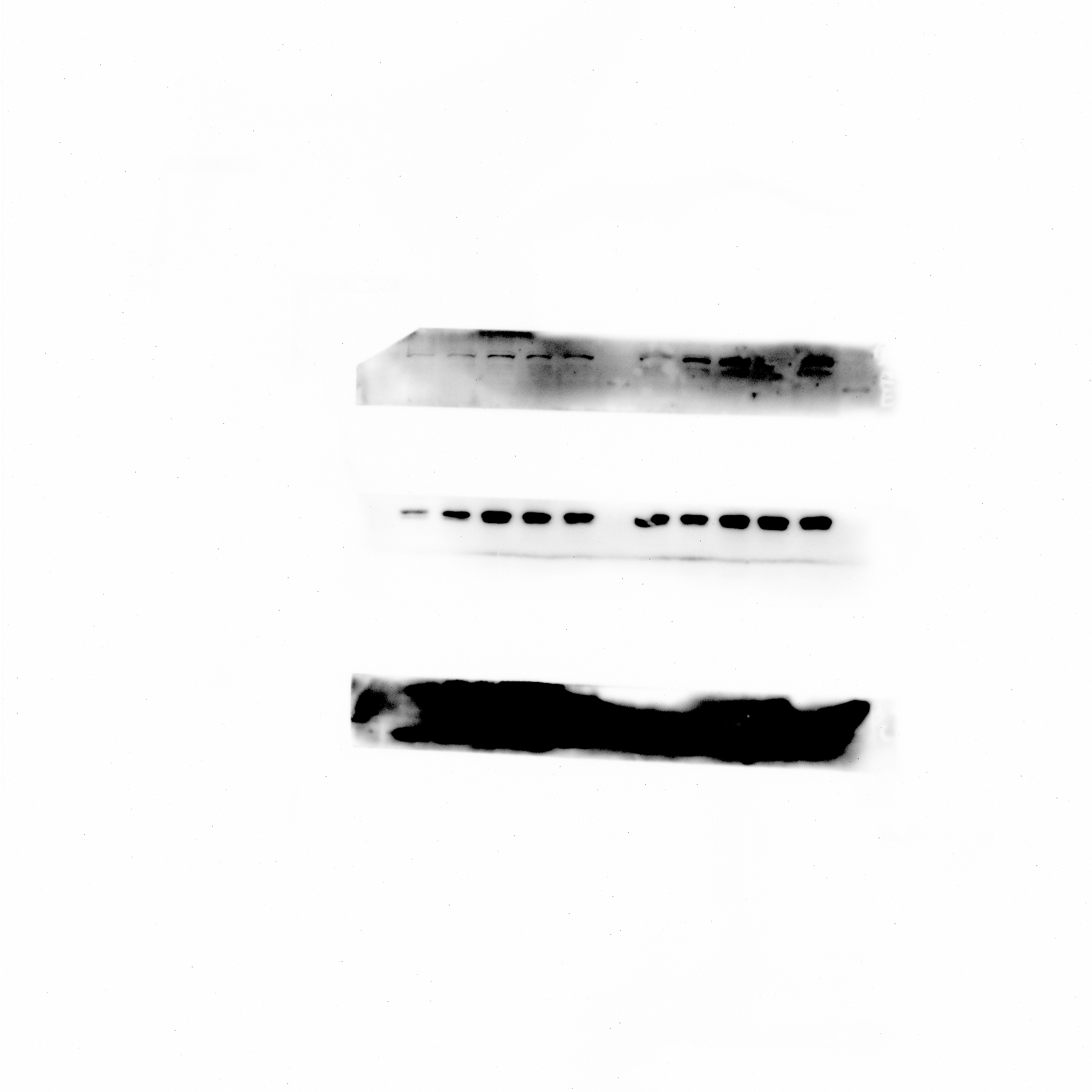

Supplement: Supplementary file 7 [file Image_6.TIF]

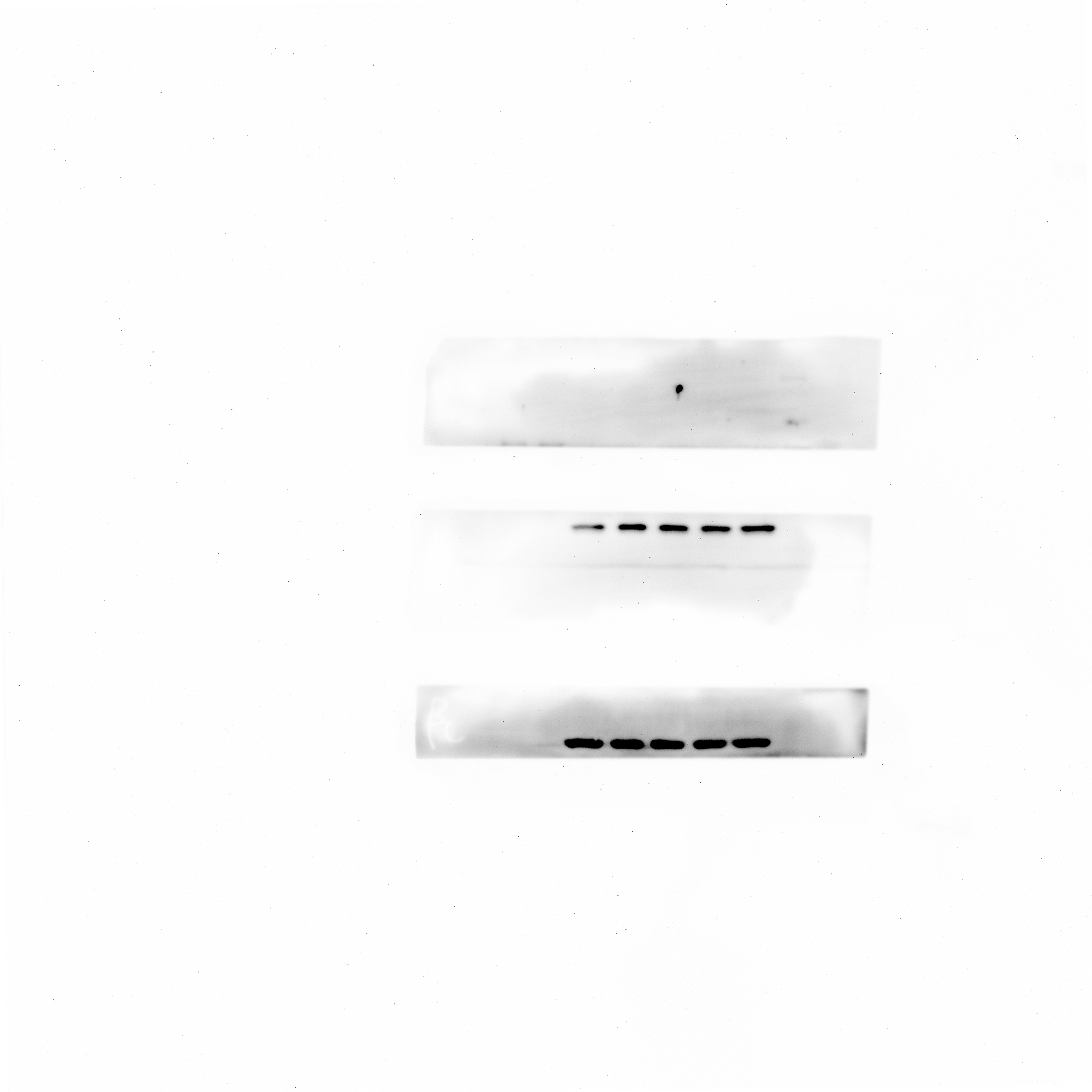

Supplement: Supplementary file 8 [file Image_7.TIF]

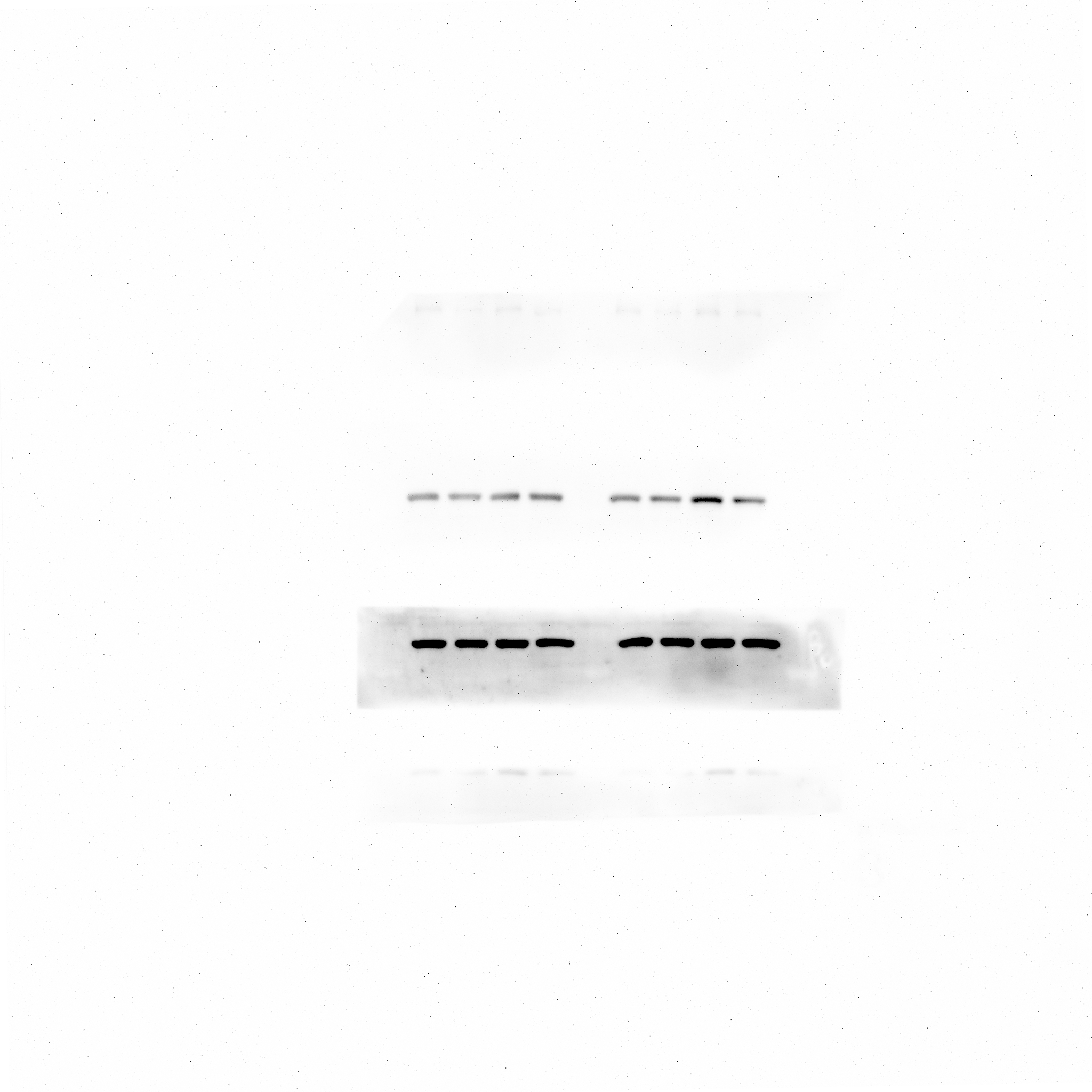

Supplement: Supplementary file 9 [file Image_8.TIF]
